# Physeal Growth Following In Situ Pinning for Slipped Capital Femoral Epiphysis and Hypothyroid Treatment: A Case Report

**DOI:** 10.7759/cureus.73214

**Published:** 2024-11-07

**Authors:** Jesse M Galina, Sawyer D Miller, Kevin T Schauer, Lauren M Harte, Michael R Ferrick

**Affiliations:** 1 Orthopaedic Surgery, Maimonides Medical Center, Brooklyn, USA; 2 Medical Student Research, Eastern Virginia Medical School, Norfolk, USA; 3 Orthopaedic Surgery, UBMD (University of Buffalo Medical Group) Orthopaedics and Sports Medicine, Buffalo, USA; 4 Pediatric Orthopaedics, UBMD (University of Buffalo Medical Group) Orthopaedics and Sports Medicine, Buffalo, USA

**Keywords:** epiphyseal plate, hypothyroidism, in situ pinning, puberty, slipped capital femoral epiphysis

## Abstract

Slipped capital femoral epiphysis (SCFE) is a common adolescent hip condition, most often seen during periods of rapid bone growth. Deficiency in thyroid hormone levels can lead to reduced bone turnover and altered epiphyseal plate activity, which may influence the outcome of SCFE pinning and other orthopedic interventions crossing the epiphysis of the femur. Our patient was a 12-year-old female child with a two-month history of atraumatic right hip pain who presented with bilateral slipped capital femoral epiphysis (SCFE) and underwent successful bilateral in situ pinning. An endocrinology workup revealed untreated hypothyroidism and the patient was started on thyroid replacement. Ten months later, X-rays revealed migration of the screws and the patient required bilateral screw removal and replacement. Patients with previously untreated hypothyroidism at the time of SCFE pinning should be monitored closely with routine X-rays until adequate stabilization of thyroid hormone levels.

## Introduction

Slipped capital femoral epiphysis (SCFE) is a common adolescent hip condition, most often seen during the teenage years, marked by periods of rapid bone growth [1]. SCFEs occur when the proximal femoral epiphysis slips posteriorly, resulting in pain and limited hip motion. Prompt identification and treatment help to prevent potential long-term complications such as avascular necrosis and early hip osteoarthritis [2].

Often multi-factorial, risk factors for SCFE include obesity, femoral retroversion, and, less commonly, hormone imbalances [3]. Loder et al. studied 85 SCFE patients with endocrinopathies and found that 40% had hypothyroidism, 25% had growth hormone deficiency, and 35% had other conditions, such as panhypopituitarism and hyperparathyroidism [4].

Hypothyroidism, characterized by inadequate thyroid hormone production, has been associated with altered bone metabolism and delayed skeletal development [5]. Thyroid hormones are crucial in maintaining bone growth, maturation, and mineralization [6]. Thyroid hormone deficiency can lead to reduced bone turnover and altered epiphyseal plate activity [7], potentially influencing the outcome of orthopedic interventions crossing the epiphysis, such as SCFE pinning. We present a case of a young female child with untreated hypothyroidism presenting with the resumption of physeal growth after undergoing bilateral screw fixation and thyroid hormone replacement.

## Case presentation

**Figure 1:**
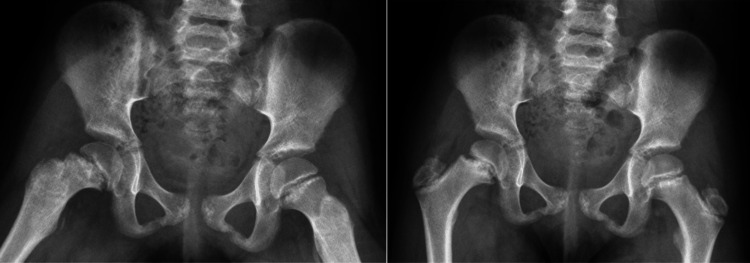
Anterioposterior and frog leg X-rays show a chronic, stable right SCFE with widening left physis in an overall skeletally immature female patient (open triradiate cartilages). SCFE: slipped capital femoral epiphysis

A 12-year-old female child presented with a two-month history of atraumatic right hip pain that she described as “achy” and extending to the thigh. There was pain with active and passive motion, restricted flexion, and external rotation. Height, weight, and BMI were 128.8 cm, 27.1 kg, and 16.34 kg/m^2^, respectively. The patient's initial X-rays are shown in Figure 1, and she was diagnosed with bilateral SCFE. Due to the appearance of her growth plates, the decision was made to order an endocrinology work-up and consultation.

**Figure 2:**
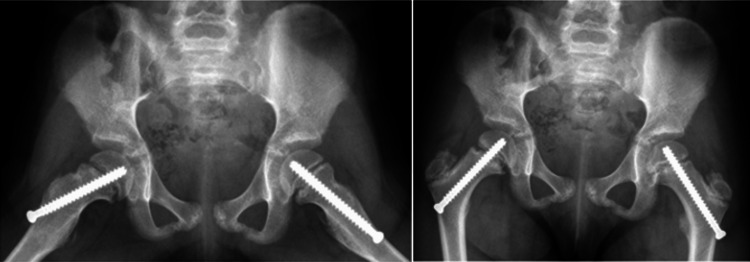
Anterioposterior and frog leg X-rays after initial bilateral pinning showing adequate screw placement.

**Figure 3:**
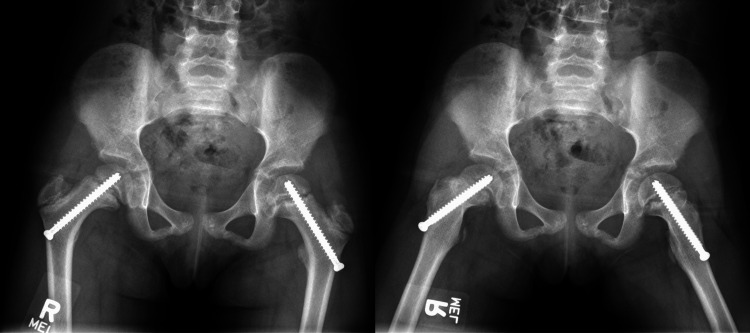
Anterioposterior and frog leg X-rays at the four-month follow-up demonstrating mild epiphyseal migration and growth along cannulated screws.

She underwent successful bilateral SCFE pinning. The decision to prophylactically pin her left hip was determined by the surgeon based on her clinical history and radiographs. Immediate postoperative X-rays are shown in Figure 2. Initial screws were placed with five threads across the physis. At the four-month follow-up, routine X-rays showed mild epiphyseal migration and growth along the bilateral cannulated screws (Figure 3).

Given abnormal thyroid labs at presentation, an endocrinology consultation was obtained. History and physical revealed several years of fatigue, long-standing constipation, dry skin, and lack of growth for one to two years. Blood work showed a thyroxine (T4) of 1.3 mcg/dL, triiodothyronine (T3) of 54 ng/dL, and thyroid-stimulating hormone (TSH) of 4.2 mU/L, and was subsequently placed on levothyroxine (Table 1).

**Table 1:** Laboratory values for patient thyroid function at presentation. T3: triiodothyronine; T4: thyroxine; TSH: thyroid-stimulating hormone

Lab test	Lab values at presentation	Lab values at 10-month follow-up	Reference range [8]
Total T4	1.3 mcg/dL	8.7 mcg/dL	4.6-11.2 mcg/dL
Total T3	54 ng/dL	n/a	75-195 ng/dL
TSH	4.2 mU/L	.83 mU/L	0.4-4.5 mU/L

**Figure 4:**
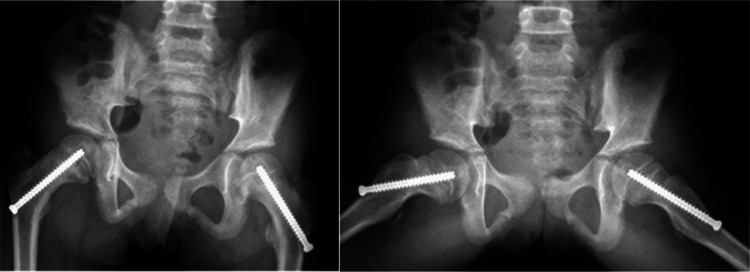
Anterioposterior and frog leg X-rays at the 10-month follow-up demonstrating complete epiphyseal migration and growth along cannulated screws.

**Figure 5:**
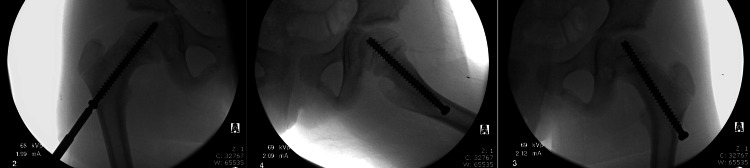
Intraoperative fluoroscopic images of screw replacement and postoperative films.

**Figure 6:**
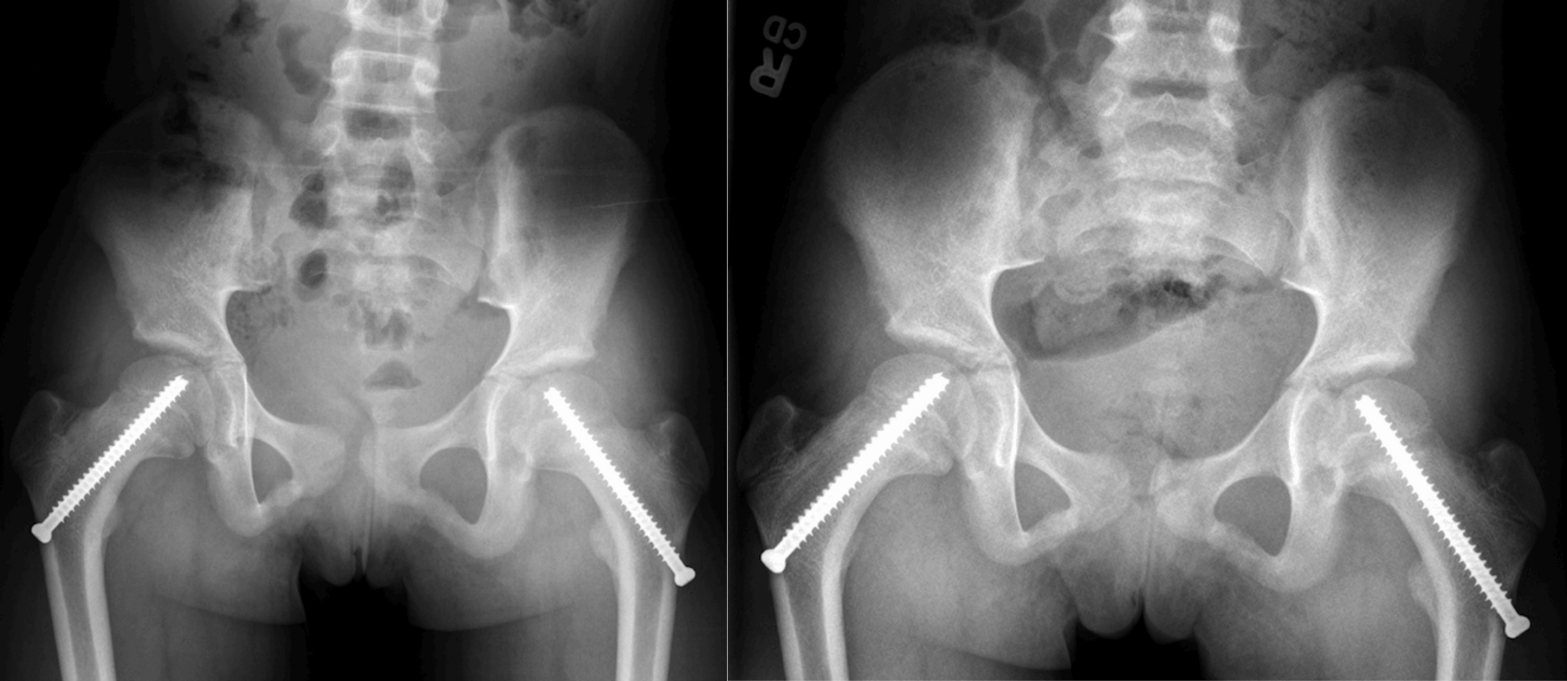
Anterioposterior and frog leg X-rays at the six-month follow-up after bilateral screw replacement. X-rays show adequate screw position and closing of growth plates.

At a 10-month follow-up visit, anterioposterior and frog X-rays showed migration of the screws, now standing with two threads across the physis (Figure 4). Total T4 increased to 8.7 mcg/dL at the 10-month follow-up, indicating an improvement in T4 levels to within the normal range with levothyroxine treatment (Table 1). The patient was indicated for removal and replacement of the bilateral screws, which was successfully completed across the same axis and without complication (Figure 5). At the six-month follow-up, the patient had no complaints, and final X-rays showed no migration of screws and evidence of closing down of the physis (Figure 6).

## Discussion

Atypical SCFE patients often present with short stature, are underweight, or are either older or younger than the average presentation of typical SCFE patients [9]. Risk factors include endocrinopathies such as hypothyroidism [4,7], vitamin D deficiency, and growth hormone abnormalities, including recombinant growth hormone therapy [9]. Therefore, many recommend endocrine screening with TSH, parathyroid hormone (PTH), and a comprehensive metabolic panel in atypical SCFE patients with any of these presentations [10,11]. If diagnosed with hypothyroidism, patients should promptly be placed on thyroid hormone replacement to ensure adequate physeal growth and healing. Treatment for SCFEs is percutaneous in situ pinning with one or two cannulated screws. In patients with unilateral SCFEs, prophylactic fixation of the contralateral screw is controversial. Currently, indications for prophylactic pinning are high-risk patients, including younger patients and those with endocrine disorders [12].

Hypothyroidism, the most common presentation of atypical SCFEs, can weaken the physis, allowing for easy slippage of the femoral head. Clinical symptoms of hypothyroidism include lethargy, fatigue, dry skin, hair loss, or constipation. Chronic hypothyroidism patients can also present with delayed bone age due to inhibition of skeletal maturation [7,13]. However, bilateral SCFEs can be the only presenting symptom in untreated hypothyroid patients [14]. In the present case of bilateral SCFE pinning in an untreated hypothyroid patient, the patient subsequently "grew off" her screws after normalizing thyroid hormone levels.

Few studies have looked at the continued epiphyseal growth after treatment in hypothyroid patients. In our case, 10 months after surgery, radiographs showed bilateral epiphyseal plate migration, showing one screw thread across the physis. Walter et al. presented a similar case in a 13-year-old hypothyroid patient who “grew off” their screws after being treated with thyroid replacement therapy [15]. At 12 months postoperatively, no screw threads crossed the physis after having three crosses the physis after the initial surgery. Ultimately, their patient required bilateral screw revision. Sleth et al. studied the continued physeal growth in patients less than 12 years old with a single cannulated screw [16]. They reported that the number of threads crossing the growth plate significantly decreased from 3.3 to 1.8 from the immediate postoperative period to an average follow-up of 2.4 years. They also found that the neck shaft angle significantly decreased over time but remained within normal limits.

## Conclusions

Hypothyroidism may have significant impacts on orthopedic interventions and as such requires prompt treatment and close monitoring of thyroid hormone levels for improved outcomes. Due to the capacity for continued physeal growth in patients with hypothyroidism, SCFE pinning should be monitored closely with routine X-rays. Depending on the amount of growth remaining, patients can be counseled that an implant exchange may be necessary after stabilizing thyroid hormone levels. This case highlights the remaining capacity for continued physeal growth and epiphyseal migration in hypothyroid patients undergoing SCFE pinning prior to the start of hypothyroid treatment.

## References

[1] Gholve PA, Cameron DB, Millis MB (2009). Slipped capital femoral epiphysis update. Curr Opin Pediatr.

[2] Kennedy JG, Hresko MT, Kasser JR (2001). Osteonecrosis of the femoral head associated with slipped capital femoral epiphysis. J Pediatr Orthop.

[3] Weiner D (1996). Pathogenesis of slipped capital femoral epiphysis: current concepts. J Pediatr Orthop B.

[4] Loder RT, Wittenberg B, DeSilva G (1995). Slipped capital femoral epiphysis associated with endocrine disorders. J Pediatr Orthop.

[5] Gouveia CH, Miranda-Rodrigues M, Martins GM, Neofiti-Papi B (2018). Thyroid hormone and skeletal development. Vitam Horm.

[6] Williams GR, Bassett JH (2018). Thyroid diseases and bone health. J Endocrinol Invest.

[7] Kadowaki S, Hori T, Matsumoto H (2017). Prepubertal onset of slipped capital femoral epiphysis associated with hypothyroidism: a case report and literature review. BMC Endocr Disord.

[8] Ross D, Cooper D, Mulder J (2023). Laboratory assessment of thyroid function. UpToDate.

[9] Blethen SL, Allen DB, Graves D, August G, Moshang T, Rosenfeld R (1996). Safety of recombinant deoxyribonucleic acid-derived growth hormone: the National Cooperative Growth Study experience. J Clin Endocrinol Metab.

[10] Lindgren AM, Lieber AM, Shah SA, Thacker MM (2023). Management of atypical slipped capital femoral epiphysis: current concept review. J Pediatr Orthop B.

[11] Loder RT, Starnes T, Dikos G (2006). Atypical and typical (idiopathic) slipped capital femoral epiphysis. Reconfirmation of the age-weight test and description of the height and age-height tests. J Bone Joint Surg Am.

[12] Shaw KA, Shiver AL, Oakes T, Fletcher ND (2022). Slipped capital femoral epiphysis associated with endocrinopathy: a narrative review. JBJS Rev.

[13] Gutch M, Philip R, Philip R, Toms A, Saran S, Gupta KK (2013). Skeletal manifestations of juvenile hypothyroidism and the impact of treatment on skeletal system. Indian J Endocrinol Metab.

[14] Witbreuk M, van Kemenade FJ, van der Sluijs JA, Jansma EP, Rotteveel J, van Royen BJ (2013). Slipped capital femoral epiphysis and its association with endocrine, metabolic and chronic diseases: a systematic review of the literature. J Child Orthop.

[15] Walter RP, Jeffery RS, Holroyd B (2013). Bilateral epiphyseal migration following fixation for slipped capital femoral epiphyses in a hypothyroid child. Acta Orthop Belg.

[16] Sleth C, Bauzou F, De Cristo C (2022). Is there a persistent capital femoral epiphysis growth after screw fixation for slipped capital femoral epiphysis?. J Hip Preserv Surg.

